# Pharmacological Treatment in Forensic Psychiatry—A Systematic Review

**DOI:** 10.3389/fpsyt.2019.00963

**Published:** 2020-01-16

**Authors:** Katarina Howner, Peter Andiné, Göran Engberg, Emin Hoxha Ekström, Eva Lindström, Mikael Nilsson, Susanna Radovic, Monica Hultcrantz

**Affiliations:** ^1^ Department of Clinical Neuroscience, Centre of Psychiatry Research, Karolinska Institutet, Stockholm, Sweden; ^2^ Department for Forensic Psychiatry, National Board of Forensic Medicine, Stockholm, Sweden; ^3^ Department for Forensic Psychiatry, National Board of Forensic Medicine, Gothenburg, Sweden; ^4^ Centre for Ethics, Law and Mental Health, Department of Psychiatry and Neurochemistry, Institute of Neuroscience and Physiology, The Sahlgrenska Academy at University of Gothenburg, Gothenburg, Sweden; ^5^ Forensic Psychiatric Clinic, Sahlgrenska University Hospital, Gothenburg, Sweden; ^6^ Department of Physiology and Pharmacology, Karolinska Institutet, Stockholm, Sweden; ^7^ Swedish Agency for Health Technology Assessment and Assessment of Social Services (SBU), Stockholm, Sweden; ^8^ Department of Neuroscience, Psychiatry, Uppsala University, Uppsala, Sweden; ^9^ Department of Philosophy, Linguistics, Theory of Science, University of Gothenburg, Gothenburg, Sweden

**Keywords:** forensic psychiatric care, mentally disordered offenders, pharmacological treatment, systematic review, antipsychotics

## Abstract

**Background:** Pharmacological treatment is of great importance in forensic psychiatry, and the vast majority of patients are treated with antipsychotic agents. There are several systematic differences between general and forensic psychiatric patients, e.g. severe violent behavior, the amount of comorbidity, such as personality disorders and/or substance abuse. Based on that, it is reasonable to suspect that effects of pharmacological treatments also may differ. The objective of this systematic review was to investigate the effects of pharmacological interventions for patients within forensic psychiatry.

**Methods:** The systematic review protocol was pre-registered in PROSPERO (CRD42017075308). Six databases were used for literature search on January 11, 2018. Controlled trials from forensic psychiatric care reporting on the effects of antipsychotic agents, mood stabilizers, benzodiazepines, antidepressants, as well as pharmacological agents used for the treatment of addiction or ADHD, were included. Two authors independently reviewed the studies, evaluated risk of bias and assessed certainty of evidence using Grading of Recommendations Assessment, Development and Evaluation (GRADE).

**Results:** The literature search resulted in 1783 records (titles and abstracts) out of which 10 studies were included. Most of the studies included were retrospective and non-randomized. Five of them focused on treatment with clozapine and the remaining five on other antipsychotics or mood stabilizers. Five studies with a high risk of bias indicated positive effects of clozapine on time from treatment start to discharge, crime-free time, time from discharge to readmission, improved clinical functioning, and reduction in aggressive behavior. Psychotic symptoms after treatment were more pronounced in the clozapine group. Mainly due to the high risk of bias the reliability of the evidence for all outcomes was assessed as very low.

**Conclusion:** This systematic review highlights the shortage of knowledge on the effectiveness of pharmacological treatment within forensic psychiatry. Due to very few studies being available in this setting, as well as limitations in their execution and reporting, it is challenging to overview the outcomes of pharmacological interventions in this context. The frequent use of antipsychotics, sometimes in combination with other pharmacological agents, in this complex and heterogeneous patient group, calls for high-quality studies performed in this specific setting.

## Introduction

### Rationale

In most jurisdictions mentally disordered offenders are given special treatment within the legal system. A criminal offender who due to a mental disorder is judged as not accountable (or legally insane) is generally considered as not responsible for his offence and hence not sentenced to a criminal sanction. In most cases such a person is, if the crime is severe and there is a great need for psychiatric care, transferred to compulsory psychiatric care (1, 2). Sweden, is one of a few exceptions, here all criminal offenders are considered responsible for their actions and forensic psychiatric care is a criminal sanction. Regardless of criminal law regulations, compulsory psychiatric care for mentally disordered offenders is often given in special forensic psychiatric institutions. Most patients have committed violent offences, leading to a potentially high risk of aggression and violent behavior within the care institution. As a result, the staffing levels in forensic wards are high compared to general psychiatric in-patient care. These circumstances result in high care costs, and forensic psychiatry often represents a large part of the overall psychiatry budget, while the population is rather small in terms of the number of patients (3).

There are a number of systematic differences between the forensic psychiatric patients and the general psychiatric patients. Firstly, forensic patients are not only patients but also offenders, in many cases offenders of severe violence (4). Even though violent behavior in an acute non-forensic setting is frequently occurring, in the forensic setting many patients have a history of more severe violence and disruptive behavior (5). Therefore, managing aggression and violent behavior are of special concerns here. The responsible clinician must not only consider the patients’ well-being, but also bear in mind the protection of society from potentially violent mentally disordered offenders (6). Secondly, in the forensic setting, all patients are involuntarily admitted and under compulsory treatment which could affect the process of shared decision making and compliance with treatment over time. Even if compulsory care also appears in general psychiatry, the majority of the psychiatric patients is under voluntary treatment. A third difference is the length of inpatient care, since in forensic psychiatric care the duration often lasts years compared to the general psychiatric care, in which the duration of inpatient care mainly is days to months. Another difference is that the proportion of comorbidity is higher compared to general psychiatry (7, 8). Psychotic disorders are the most frequent primary diagnosis in this population, but comorbidity such as personality disorders, substance abuse and neuropsychological disorders, are more common here, compared to in general psychiatry. In the light of these differences it is plausible that effects of pharmacological treatments could differ between the forensic and the general psychiatric setting.

The vast majority of patients in forensic psychiatry are treated with pharmacological agents (9). Psychotic disorders are well represented within the forensic psychiatric population, and pharmacological treatment with antipsychotics is crucial in this regard (10, 11). When comparing the use of traditional versus atypical antipsychotics in Sweden, we found that it was more common to use traditional antipsychotics in the forensic setting and also that combinations of several antipsychotic agents were more frequent (12). A few systematic reviews relevant to the field have been published. A recent review (13) emphasizes the value of clozapine in the treatment of violent and aggressive behaviors also in a forensic population. Another recent study (14) identified two previously published systematic reviews, which in part addressed the effects of pharmacological interventions within forensic psychiatry (15, 16), showing that only a few primary studies had been published, all of which were assessed as being at high risk of bias. However, the reports had methodological limitations and could potentially have resulted in relevant records having been overlooked. Also, and more importantly, as the last literature searches were performed in 2010 (16) and 2012 (15) respectively, an update is needed to ensure that research published over the previous six years is included.

Based on the fact that the general and forensic psychiatric populations differ in many respects, it is reasonable to suspect that effects of pharmacological treatments also may differ. Since there may be relevant studies from the forensic setting published since the latest systematic reviews in the field, we aimed to perform an updated systematic review, searching for pharmacological intervention studies in the specific forensic psychiatric setting.

### Objective

The aim of this systematic review was to investigate therapeutic effects and side effects of pharmacological treatment within forensic psychiatry, with a focus on outcomes important to patients as well as society. When searching for relevant literature we chose to focus specifically on forensic psychiatric patients with psychotic disorders, personality disorders, autism spectrum disorders and substance use disorders, as these are the most common diagnoses in this setting.

## Materials and Methods

### Protocol and Registration

This systematic review was conducted at The Swedish Agency for Health Technology Assessment and Assessment of Social Services (SBU), and a Swedish version was submitted to the Swedish Ministry of Social Affairs (www.sbu.se/258) in June 2018. A peer-reviewed protocol, including pre-specified objectives, was registered in PROSPERO (http://www.crd.york.ac.uk/PROSPERO/display_record.php?ID=CRD42017075308). The systematic review process follows the general concepts covered by PRISMA (17).

### Eligibility Criteria

We included studies of adult offenders (over 15 years of age, since that is the cut off for criminal responsibility in Sweden) with severe mental disorder, including psychotic disorders, autism spectrum disorders or personality disorders, in forensic psychiatric care. The interventions were antipsychotic agents, mood stabilizers, benzodiazepines and benzodiazepins-like agents, pharmacological addiction treatment, pharmacological ADHD-treatment, and antidepressants. All primary studies with a control group were included without restrictions as to study type. We tried to include a wide range of different outcomes: symptoms of psychosis (clinical functioning), aggression and violent behavior, adverse events, mortality, reoffending (both violent acts and non-violent offences), mental and physical health, time outside the hospital before readmission, quality of life, and long-term compliance to treatment.

### Search Strategy

The literature search was performed by an information specialist and included the databases Cinahl, Cochrane, EMBASE, PsycINFO, PubMed, and Scopus. The search covered studies published in English, Swedish, Norwegian or Danish up to January 11, 2018. In addition, references from narrative reviews and articles published in international journals, not identified in the main search, were also included. This by going through reference list from the articles found in the main search. Grey literature, such as conference abstracts or dissertations, were excluded. Electronic searches were conducted using Medical Subject Headings (MeSH) and relevant text word terms. The detailed search strategy can be found in **Supplementary Material**.

### Study Selection

Two reviewers independently screened the titles and abstracts identified by the search strategy. All studies of potential relevance according to the inclusion criteria were obtained in full text and two reviewers independently assessed them for inclusion. Any disagreement was resolved by discussions, with involvement of a third review author, when necessary.

### Risk of Bias in Individual Studies

Two reviewers independently assessed the risk of bias with the use of tools developed for randomized and non-randomized controlled trials, including signaling questions to address selection bias, performance bias, detection bias, reporting bias and bias due to conflicts of interest. The tools for assessing risk of bias in individual studies were developed at SBU (18) and focus on the same aspects that are included in international guidelines for reporting standards (19, 20). Before starting the assessments, the questions were thoroughly discussed so that all reviewers had a common understanding as to how these criteria could affect the results in this specific research area. Each study was rated as having a low, moderate or high risk of bias.

### Data Collection Process

From each included study data were extracted and inserted into tables by one reviewer, followed by auditing of the data extraction by another reviewer. Any disagreement was resolved by discussion. Information concerning study design, setting, population, intervention, control group, outcome and results were extracted from each included study.

### Data Analysis

We anticipated that there would be limited scope for meta-analysis because of the range of different outcomes measured across the small number of existing trials. However, if studies would have used the same type of intervention and comparator, with equal outcome measures, we would have pooled the results using a random-effects meta-analysis. The certainty of the evidence for each outcome was assessed using Grading of Recommendations Assessment, Development and Evaluation (GRADE) (21) and we followed the suggested criteria for using GRADE presented on the GRADE working group website (www.gradeworkinggroup.org).

## Results

### Study Selection and Characteristics

**Figure 1:**
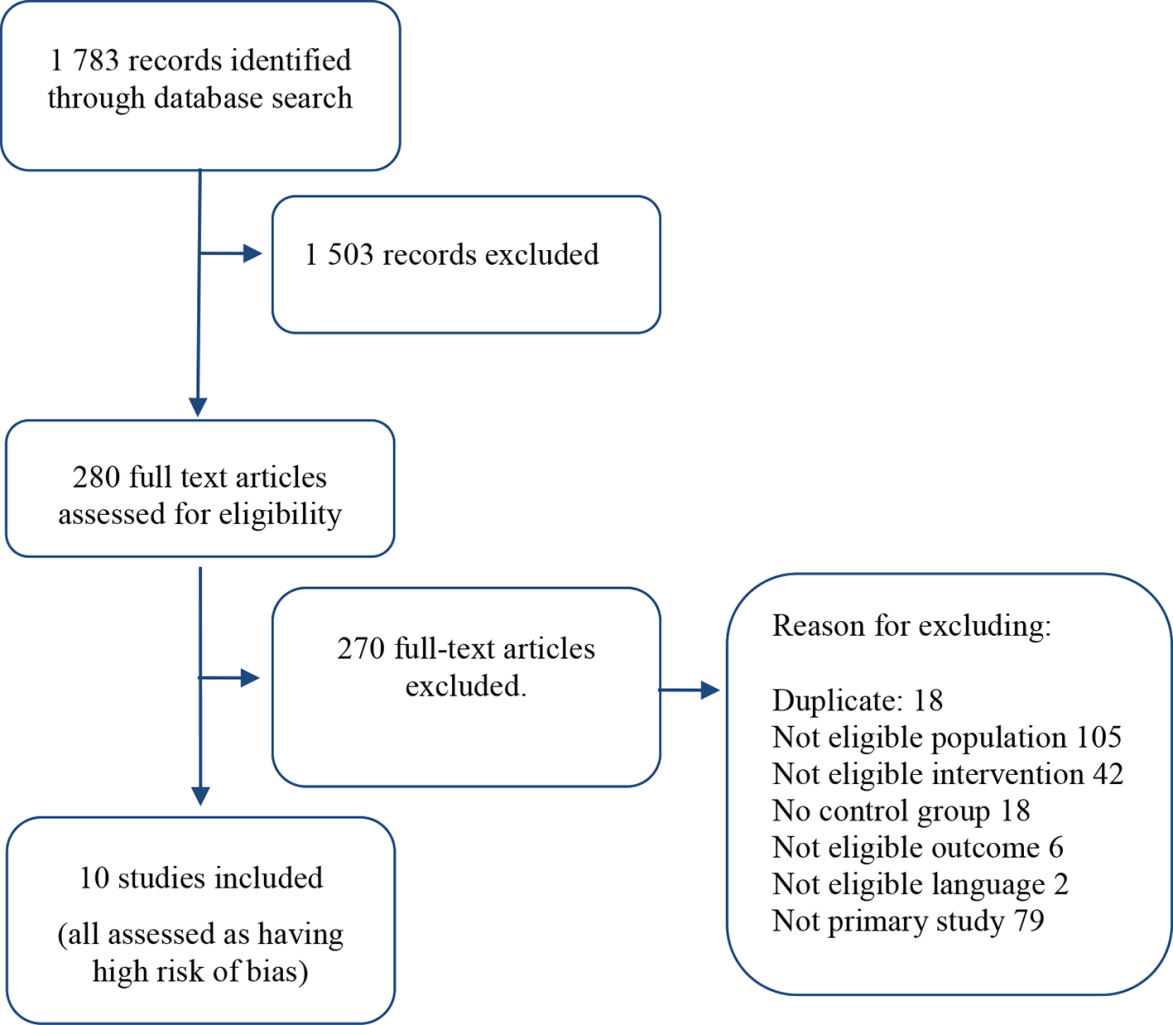
Flowchart over literature search.

The systematic literature search resulted in 1783 records (titles and abstracts), out of which 10 studies fulfilled the inclusion criteria. The primary reasons for exclusion of studies were that the studied populations were not treated within a forensic setting or the studies did not evaluate the effect of an intervention (list of excluded full-text studies with reasons for exclusion are available on request). All 10 included studies (22–31) were assessed as having a high risk of bias, primarily due to selection bias. The review process and number of reviewed articles are summarized in the flow diagram in **Figure 1**.

### Synthesized Findings

The 10 included studies, which are further described in **Table 1**, were conducted in the United Kingdom (24, 25, 30, 31), USA (23, 29), and Canada (22, 26–28). Seven of the studies were retrospective. The study populations were exclusively from high security hospitals. One study followed the patients from in- to outpatient care (28). Half of the studies investigated the specific anti-psychotic clozapine with regard to effect on aggressive behavior, psychotic symptoms, time of treatment inside the hospital before release and time outside the hospital before readmission (22, 25, 28–30).

**Table 1:** Characteristics of included studies.

Study	Country and settings	Type of study	Population	Intervention	Control	Outcome	Results	Strenghts and limitations
Balbuena, et al. (22).	Canada Forensic psychiatric hospital, dedicated to high-risk, high-need, federally sentenced (2 years or more) mentally disordered offenders.	Retrospective study	Clozapine group: n = 65, mean age 34 (63 male, 2 female). Control group: n = 33, mean age 37 (31 male, 2 female). All patients and controls had psychosis or related disorders according to DSM-IV. No information on co-morbidity.	Clozapine treatment for 6 months. Dose or administration form is not given.	Treatment with traditional antipsychotics at the same hospital for 6 months	Frequency of noncompliant incidence, change in BPRS total score. Institutional pay was recorded as a measure of good behavior, presumably reflecting clinical functioning.	Clozapine-group: Mean pay increase: 38/65 got increased pay level Mean BPRS score: 38.5 Mean number of post-treatment offences during 12 months: 0.62 Control group: Mean pay increase: 10/33 got increased pay level Mean BPRS score: 30.4 Mean number of post-treatment offences during 12 months: 1.37	High risk of bias **Comment**: Non-randomized., risk of selection bias. The effect of clozapine may be under-estimated because of negative selection (all patients in the clozapine group were non-responders to traditional antipsychotics). Lack of compliance analysis in the clozapine group in contrast to the control group. Number of interactions were not recorded during the six-month treatment period, but during the six-month period after. Unclear whether patients during the latter six-month period were on clozapine or other antipsychotics.
Beck et al. (23)	USA Three forensic treatment wards at state mental hospital.	Retrospective study	Risperidone group: n = 10, mean age 39 (all male). Controls: n = 10, mean age 40 (all male). All patients and controls had schizophrenia or schizoaffective disorders according to DSM-IV. No information on co-morbidity.	Risperidone treatment for 6 + 6 months, 6 mg/day, administration form not given.	Treatment with traditional antipsychotics (equivalent with 2,000 units of chlorpromazine) at the same hospital for 6 + 6 months.	Scores on TSBC, reflecting clinical functioning. Frequency counts of aggressive behaviour and bizarre motor behaviours were recorded.	No difference between risperidone patients and controls with regard to overall clinical functioning or aggressive behaviour.	High risk of bias **Comment:** Non-randomized and risk of bias since patients were recruited from 3 different wards. Lack of information in the control group. Only the frequency of aggressive behaviour was recorded. In the control group only chlorpromazine equivalents were given instead of a detailed description of what antipsychotics used.
Collins et al. (24)	UK High security hospital	Randomized single-blind trial	Lithium add-on: n = 21, mean age 39 (all male). Controls: n = 22, mean age 38 (all male). All patients and controls had schizophrenia or related disorders according to DSM-III. No information on co-morbidity.	Addition of lithium carbonate to traditional antipsychotics 400 mg twice daily	Treatment with various antipsychotics throughout the treatment period.	Psychiatric conditions according to the Manchester Scale (modified to separate flattening and incongruity of affect) and SANS Scale.	Lithium add-on showed no improvement in psychiatric condition	High risk of bias **Comment:** High drop-off in the treatment group. Individual antipsychotic treatment was not reported.
Dalal et al. (25)	UK High security hospital	Retrospective study.	Clozapine group: n = 50 (44 male, 6 female). Information on age not given, unless that mean age at first psychiatric contact was 20 years. Control group: n = 50 Schizophrenia or schizoaffective disorder. Local rating scales of positive symptoms were used.	Clozapine treatment baseline before, after 6 months, after 1 year, after 2 years. No specific doses of clozapine is given, only, in some cases, chlorpromazine-equivalent doses.	No control group, effects were compared with baseline values. 50 non-clozapine patients from the same hospital were used as controls, but for the comparison of discharge rate only.	Frequency of violence and self harm. Discharge rate from the hospital Positive symptoms according to Health of the Nation Outcome Scales	50% of patients showed a reduction in positive symptoms and aggressive behaviour after 2 years of treatment. Significant increase in discharge rate in patients that continued treatment compared to those that discontinued clozapine. However, this discharge rate was not higher compared to the controls.	High risk of bias **Comment:** Non-randomized. No internationally accepted rating scales were used. Negative symptoms were not recorded. Thus, unclear patient population. Missing information of the control group.
Gobbi et al. (26)	Canada High security	Randomized open-label study.	Quetiapine group: n = 8, mean age 43 (7 male, 1 female). Olanzapine group: n = 7, mean age 38 (all male). Patients were diagnosed with schizophrenia, schizoaffective disorder, or paranoid disorder (DSM-IV)	Comparison between Quetiapine treatment (10 weeks, mean dose 475 mg/day) and Olanzapine treatment (10 weeks, mean dose 15 mg/day)	Comparison between quetiapine and olanzapine Two different treatment groups.	Impulsive and aggressive behaviour according to “Modified Overt Aggression Scale” and “Impulsivity Rating Scale” Psychotic symptoms according to BPRS, PANSS, and CGI.	Both drugs decreased impulsivity and psychotic symptoms. No significant difference between the drugs were observed. Quetiapine was better than olanzapine in improving depression symptoms.	High risk of bias. **Commen**t: Sponsored study. High quality of the study, although very small sample size. Vague information on medication at baseline (day 0 prior to treatment).
Gobbi et al. (27)	Canada High security	Retrospective study	Topiramate group: n = 16, mean age 37 (34 male, 3 female). Valproate group: n = 16, mean age 39 (all male). Combination group: n = 13, mean age 41 (12 male, 1 female) Patients were diagnosed with schizophrenia, schizoaffective disorder, any subtype of delusional disorder, or bipolar disorder	Add-on treatment (8-12 weeks) to traditional antipsychotics with topiramate (mean dose 250 mg/day), valproate (dose corresponding to plasma concentration of 700 µM), or a combination of both drug.	Three different treatment groups.	Aggression (Overt Aggression Scale), agitation (Agitation-Calmness Evaluation Scale), psychotic episodes (BPRS), number of therapeutic isolation and surveillance interventions.	All groups showed a reduction in agitation, aggressive behaviour. Valproate group and the combination of topiramate and valproate showed a reduction in psychotic episodes.	High risk of bias **Comment**: Non-randomized. Only part of the data was analysed in a blind manner. A selection of patients was made - only patients being able to tolerate topiramate/valproate were chosen. Greater loss of patients in topiramate-group.
Mela and Depiang (28)	Canada Open care patients from Regional Psychiatric Center	Retrospective study	Clozapine treatment: n = 41, Non-clozapine: n = 21 Age or gender not given. Offenders with mental disorders	Clozapine treatment more than 6 weeks in open care, dose titrated to therapeutic relevance. 2-years follow up.	Treatment with antipsychotics other than clozapine	Number of reoffending behavior (nonviolent, violent, and sexual). Time from release to the first offence. Crime-free time.	The clozapine group had a lower, although non-significant, incidence of all of the categories of reoffending, except sexual Time from release to first offence longer in the clozapine group. Crime-free time longer in the clozapine group.	High risk of bias **Comment:** Compliance not accounted for during the 2 years of follow-up. Contact with the health professionals may vary between the groups. Diagnosis not specified. No randomization. In the comparison group various antipsychotics were used. The effect of clozapine may be underestimated because of a negative selection (all patients in the clozapine group were non-responders to traditional antipsychotics).
Stoner et al. (29)	USA Psychiatric Rehabilitation Center-. Patients hospitalized due to forensic court commitment, security level unclear.	Retrospective study	Haloperidol treatment: n = 78 Clozapine treatment n = 21 Total sample: 69 male and 15 women Patients were diagnosed with schizophrenia or substance abuse.	Clozapine treatment Haloperidol treatment (either orally, mean dose 15.5 mg/day, or intramuscularly mean dose 206 mg every 4^th^ week).	Two treatment groups: comparison between clozapine and haloperidol	Psychiatric symptoms according to GAF Conditional release Revoked conditional release	Haloperidol group: 59% showed improved GAF scores. 33% successfully obtained conditional release. Clozapine group: 86% showed improved GAF scores. 38% successfully obtained conditional release. Periods of conditional release before revocation were longer in the clozapine group	High risk of bias **Comment**: Non-randomized. Various diagnosis, including both schizophrenia and drug abuse - groups were not homogenous. Some patients were treated by a combination of haloperidol and clozapine. Haloperidol was given either orally every day or intramuscularly every 4 weeks. The effect of clozapine may be underestimated because of a negative selection (all patients in the clozapine group were non-responders to traditional antipsychotics).
Swinton and Haddock (30)	UK High security hospital	Retrospective case-control study	Clozapine group: n = 106, mean age 29 (73 male, 33 female). Non-clozapine group: n = 106, mean age 30 (73 male, 33 female). Diagnosis: mainly schizophrenia.	Clozapine treatment	Treatment with antipsychotics other than clozapine at the same hospital for	Evaluation of discharge rates	Clozapine group achieved increased rates of discharge when compared with non- clozapine group. It took more than one year until this effect was obtained	High risk of bias **Comment:** relatively high drop-out. Some female cases in the study may not have had a diagnosis of schizophrenia. No information on the treatment of the non-clozapine group.
Tavernor et al. (31).	UK High security hospital	Retrospective case-control study	High-dose group: n = 32, mean age 39 Control group: n = 32, mean age 38 No information on gender. Diagnosis of schizophrenia (62) or schizoaffective disorder (2)	High doses of traditional antipsychotic drugs	Patients with standard doses of antipsychotic drugs	Psychiatric symptoms evaluated by BPRS, GAS, SDAS, and NOSIE	Cases had higher BPRS total score than controls, as well as neurological side-effects. Cases were rated as more aggressive than controls. Conclusion: little benefit for the use of high-dose antipsychotics.	High risk of bias **Comment:** Selection bias where cases had worse psychiatric symptoms and may have been prescribed a high-dose antipsychotic drug. Many patients received a combination of antipsychotic drugs and doses of antipsychotics were only given in equivalents of chlorpromazine. The antipsychotic drugs used are not specified. It is unclear whether each case received the same antipsychotic drug as the matched control.

In **Table 2** we present the different outcomes according to GRADE. Some of our defined outcomes, survival, compliance to treatment and quality of life, were not present in any of the selected studies. The outcomes that we did find in the included studies were psychotic symptoms, side-effects of pharmacological treatment, reoffending, time outside the hospital before readmission, clinical functioning, aggressive behavior and length of stay in hospital. When measuring psychotic symptoms after treatment, the clozapine group showed more psychotic symptoms compared to the control group (22), while there were no differences between groups studying ad-on treatment with lithium (24) or quetiapine versus olanzapine (26). In one study there were three groups with mood stabilizers, which found reduction on psychotic symptoms in the group with valproate and the combination valproate and topiramate after treatment (27). When studying crime-free time and time in the community between discharge from hospital to reoffending or readmission, two studies showed valuable effects in the clozapine group (28, 29) compared to other antipsychotics. Regarding measures of time spent in hospital after the start of treatment, two studies showed positive effects of clozapine (29, 30), while a third study did not find any differences between clozapine and other antipsychotics (25). Two studies found improvement in clinical functioning in the clozapine group compared to other antipsychotics (22, 29), while a study comparing risperidone compared to traditional antipsychotics did not find any differences (23). We found two studies showing positive effects of clozapine on aggressive episodes (22, 28), while there were no group differences when comparing risperidone with traditional antipsychotic (23), quetiapine with olanzapine (26) or topiramate with valproate (27).The only study comparing high with standard doses of antipsychotics showed more pronounced psychotic symptoms and more side effects in the high dose-group after the treatment period (31).

**Table 2:** Summary of findings according to GRADE.

Reference	Pharmacological treatsment	Outcome (Variable)	Studydesign Number of studies (Participants)	Results	GRADE assessment	Comments
Balbuena et al. (22)	Clozapine vs other antipsychotics	Symptoms of psychoses (measured with BPRS)	Non-randomized retrospective study 1 (98)	More psychotic symptoms assessed with BPRS in the clozapine group compared to other antipsychotics.	Very low certainty We are uncertain about the effect of clozapine on psychotic symptoms compared to other antipsychotic treatments	-3 risk for bias (Selection bias: non-randomized study, unclear if the two groups were comparable at onset of study)
Mela and Depiang (28)	Clozapine vs traditional antipsychotics	Crime free time (number of months from release to reoffending)	Non-randomized retrospective study 1 (62)	Time from release to reoffending was on average 52 months longer in the clozapine group	Very low certainty We are uncertain about the effect of clozapine on the length of “crime free time” compared to traditional antipsychotics	-3 risk for bias (Selection bias: non-randomized study, unclear if the two groups were comparable at onset of study) Unclear if treatment continued during time after release, the so called “crime free time”
Dalal et al. (25) Swinton and Haddock (31) Stoner et al. (29)	Clozapine vs traditional antipsychotics	In-patient time (Measured as time from treatment start until release from ward)	Non-randomized retrospective studies 3 (n = 411)	Subjects in the clozapine-group were released faster compared with subjects in traditional antipsychotic group (29, 31) In-patient time did not differ between clozapine group and other antipsychotics-group. Drop outs in the clozapine group had significant longer in-patient time. (25)	Very low certainty We are uncertain about the effect of clozapine on in-patient time before discharge compared to traditional antipsychotics	-3 risk for bias (Selections bias: non-randomized studies, unclear if the two groups were comparable at onset of study) -1 inconsistency
Stoner et al. (29) Mela and Depiang (28)	Clozapine vs other antipsychotics	Time in freedom (time on conditional release before readmission or reoffending)	Non-randomized retrospective studies 2 (n = 161)	Patients treated with clozapine had longer time in freedom before readmission compared to patients on haloperidol (29) Time from release to reoffending was 52 month longer in the clozapine group (28)	Very low certainty We are uncertain about the effect of clozapine on time on conditional release before readmission or reoffending compared to traditional antipsychotics	-3 risk for bias (Selections bias: non-randomized study, unclear if the two groups were comparable at onset of study)
Balbuena et al. (22) Mela and Depiang (28)	Clozapine vs other antipsychotics	Aggressive behavior (Aggressive episodes reported by staff or police, intensity of aggressive behavior under treatment, and time from release to first aggressive episode)	Non-randomized retrospective studies 2 (n = 160)	Patients on clozapine had fewer episodes of aggressive behavior compared to the group with other antipsychotics. (22, 28) Longer time from release to first aggressive episode in clozapine group compared to other antipsychotics (28)	Very low certainty We are uncertain about the effect of clozapine on aggressive behavior compared to traditional antipsychotics	-3 for bias (Selections bias: non-randomized studies, unclear if the groups were comparable at onset) -1 indirectness (indirect measures for aggressive behavior)
Balbuena et al. (22) Stoner et al. (29)	Clozapine vs other antipsychotics	Clinical functioning (measured with Global assessment of functioning, GAF and a reward system at the ward)	Non-randomized retrospective studies 2 (n = 197)	Rewards for good behavior were higher in clozapine group (38/65 compared to control group (10/33) (22) Improvement in GAF-scores in 86% of clozapine group compared to 59% in haloperidol group (29)	Very low certainty We are uncertain about the effect of clozapine on clinical functioning compared to traditional antipsychotics	-3 for bias (Selections bias: non-randomized study, unclear if the two groups were comparable at onset of study)
Beck et al. (23)	Risperidon vs traditional antipsychotics	Clinical functioning	Non-randomized retrospective study 1 (n = 20)	No difference in clinical functioning between risperidone and traditional antipsychotics.	Very low certainty We are uncertain about the effect of risperidone on clinical functioning, compared to traditional antipsychotics.	-3 bias (Selections bias: non-randomized study, unclear if the two groups were comparable at onset of study) -1 imprecision (non-significant results with low number of participants)
Beck et al. (23)	Risperidon vs traditional antipsychotics	Aggressive behavior (number of aggressive episodes)	Non-randomized retrospective study 1 (n = 20)	No difference in aggressive behavior between the group with risperidone and traditional antipsychotics	Very low certainty We are uncertain about the effect of risperidone on aggressive behavior compared to traditional antipsychotics	-3 bias (Selections bias: non-randomized study, unclear if the two groups were comparable at onset of study) -1 imprecision (non-significant results with low number of participants)
Collins et al. (24)	Mood stabilizer (lithium) as ad-on treatment to antipsychotics	Psychotic symptoms (measured with “Manchester Scale”)	Randomized controlled study (RCT) 1 (n = 43)	No differences in psychotic symptoms between the group receiving only antipsychotic or lithium as ad on treatment	Very low certainty We are uncertain about the effect of ad on treatment with lithium on psychotic symptoms	-2 risk for bias (treatment bias due to lack of blinding, the antipsychotic treatment was different between study groups) -1 imprecision (non-significant results with low number of participants)
Tavernor et al. (31)	High-dose vs. normal dose with traditional antipsychotic	Psychotic symptoms (measured with BPRS)	Retrospective case – control study 1 (n = 64)	Patients treated with high-dose antipsychotic showed more psychotic symptoms compared to patients in the group treated with normal dose of antipsychotics	Very low certainty We are uncertain about the effect of high dose antipsychotic on psychotic symptoms compared to standard dose	-3 risk for bias (Selection bias: non-randomized study, unclear if the two groups were comparable at onset of study)
Gobbi et al. (26)	Quetiapin vs Olanzapin	Psychotic symptoms (measured by BPRS, PANSS, CGI)	RCT 1 (n = 15)	Both quetiapin and olanzapine reduced psychotic symptoms, no difference between groups	Very low certainty We are uncertain about the comparative effects of Quetiaptin and Olanzapin. on psychotic symptoms	-2 risk for bias -1 imprecision (non-significant results with low number of participants)
Gobbi et al. (27)	Topiramate vs. Valproat vs. Combination of Topiramate & Valproat	Psychotic episodes (measured by BPRS)	Non-randomized retrospective study 1 (n = 45)	Valproat group and the combination group showed reduction in psychotic episodes	Very low certainty We are uncertain about the comparative effects of Topiramate, Valproat or a combination of both, on psychotic episodes	-3 risk for bias (Selections bias: non-randomized study, unclear if the two groups were comparable at onset of study) -1 imprecision (low number of participants)
Gobbi et al. (26)	Quetiapin vs Olanzapin	Aggressive behavior (measured with modified “Overt Aggression Scale” and “Impulsivity Rating Scale”)	RCT 1 (n = 15)	Both quetiapin and olanzapine reduced aggressive behavior, no difference between groups	Very low certainty We are uncertain about the comparative effects of Quetiaptin and Olanzapine on aggressive behavior	-2 risk for bias -1 imprecision (non-significant results with low number of participants)
Gobbi et al. (27)	Topiramate vs. Valproat vs. Combination of Topiramate & Valproat	Aggressive behavior (measured with modified “Overt Aggression Scale” and “Impulsivity Rating Scale”)	Non-randomized retrospective study 1 (n = 45)	All groups did show reduced aggressive behavior, no difference between groups	Very low certainty We are uncertain about the comparative effects of Topiramate, Valproat or a combination of both, on aggressive behavior	-3 risk for bias (Selections bias: non-randomized study, unclear if the two groups were comparable at onset of study) -1 imprecision (low number of participants)
Tavernor et al. (31)	High-dose vs. normal dose with traditional antipsychotic	Side effects (neurological and autonomic measures with side effect scale)	Retrospective case – control study 1 (n = 64)	High-dose treatment resulted in higher frequency of neurological and autonomic side effects compared to normal dose treatment	Very low certainty We are uncertain about the effect of high dose antipsychotic on side effects compared to standard dose	-3 risk for bias (Selection bias: non-randomized study, unclear if the two groups were comparable at onset of study)

### Risk of Bias

All outcomes were assessed as very low certainty due to a severe or critical risk of bias, imprecision and/or indirectness and inconsistency. There were problems with selection bias in all the included studies. As all the studies were conducted in clinical settings and only a few had randomization (24, 26), there were potential differences between comparison groups of patients. In many studies there was also a lack of transparency as to how the patients were selected (23, 27) and a lack of information about essential characteristics of the groups, such as comorbidity and substance abuse (22–24, 28, 30). Due to the non-randomized study design, as well as severe additional concerns about selection bias, the certainty of the evidence was assessed as very low.

## Discussion

The limited findings from this systematic review reveal a knowledge gap in pharmacological treatment within forensic psychiatric care. Even though the use of pharmacological agents is high in forensic psychiatric settings, these patients are seldom included in pharmacological trials. As in the two previous systematic reviews we did not find any primary studies of high quality (15, 16). Rather, all selected studies had a high risk of bias. Compared to these two previous reviews, we included four primary studies which had not previously been included. One study compared the effect of add-on treatment with lithium (24) and another compared clozapine to haloperidol (29). Two studies were published later than both of these reviews; one compared treatment with quetiapine versus clozapine (26), the other followed inpatients through to outpatient care comparing clozapine with other antipsychotics (28).

Most of the studies found were retrospective (22, 23, 25, 27–31) and performed in a clinical context, which resulted in a high risk of selection bias. Only two of them were prospective (24, 26). Half of the studies analyzed the therapeutic effects of clozapine (22, 25, 28–30). As clozapine is a third-hand choice in guidelines for treatment of psychotic disorders, all the patients receiving clozapine were non-responders to traditional antipsychotics. Potentially these patients could have been suffering from more severe psychotic symptoms, or other kinds of symptoms, resistant to traditional antipsychotics. For example, psychotic symptoms before treatment have been more pronounced in the clozapine group, indicating a selection bias (22).

Guidelines for forensic psychiatric treatment have been proposed, although, the authors found that the evidence base for forensic-psychiatric practice is weak (32). In another study presenting guidelines for aggressive psychiatric patients in California, these guidelines were developed from a collection of prescribing recommendations, clinical trial results, and years of clinical experience in treating patients who are persistently violent or aggressive in the California Department of State Hospital System, and included recommendations provided off-label prescribing of pharmacological agents (33).

We think that the specific circumstances within the forensic psychiatric care; forensic patients in general being suffering more often suffer from comorbidity as well as aggression and violent behavior and the fact that one objective of the care is coetail protection may influence the outcome of pharmacological treatment. Therefore, we maintain that there is an urgent need for studies performed in this unique context. Randomized prospective studies in forensic psychiatric samples should be prioritized. Forensic populations ought to be studied with higher precision, with regard to their particular context. Also, there are specific outcomes of special interest to forensic settings compared to the general psychiatry, such as reoffending and violent behavior. Clearly, any pharmacological treatment with a potential effect on offending and violent behavior would be of great interest not only for the patients but also society at large. Since most forensic patients are treated involuntarily for a long time period, which entails a major infringement of several human rights, interventions shortening the length of stay in hospital would also be of great ethical value. It would also be of great interest from a health economic point of view, as the cost of pharmacological treatment is almost negligible in comparison to all other costs of forensic psychiatric care.

### Limitations

This systematic review only included studies produced within the specific setting of forensic psychiatry. However, there could be studies produced in other settings, such as in general psychiatry or in correctional samples, that could have added evidence, for example those focusing on pharmacological effects on patients with comorbid conditions. Our study selection was also limited to studies written in English, Swedish, Norwegian or Danish language, and we did not include gray literature. Therefore, there is always a potential that studies only published as reports etc., or in other languages were missed. Studies which may have been published after the original literature search in January 2018 were not included in this review.

## Conclusions

This systematic review highlights the scarcity of knowledge on the effectiveness of pharmacological treatment within a forensic psychiatric population. Thus, due to the very few studies available in this setting, as well as limitations in their execution and reporting, it is challenging to overview the outcomes of pharmacological interventions in this regard. The frequent use of antipsychotics, with or without a combination of other pharmacological agents, in this complex and heterogeneous patient group, calls for high-quality studies performed in specific settings. Such strategies are also highly warranted from an ethical as well as health economics standpoint.

## Author Contributions

KH, PA, EL, GE, MH, and MN defined PICO, assessed relevance of abstracts and full text articles, extracted data, assessed the risk of bias in individual studies, analyzed and interpreted the results, and wrote the manuscript. EE and SR defined PICO, analyzed and interpreted the results, and wrote the manuscript.

## Funding

This systematic review was conducted at The Swedish Agency for Health Technology Assessment and Assessment of Social Services (SBU). The funding agency had no role in study design, data collection, data analysis, data interpretation or writing of the report.

## Conflict of Interest

The authors declare that the research was conducted in the absence of any commercial or financial relationships that could be construed as potential conflicts of interest.
