# Supplementary material for: Pharmacological Treatment in Forensic Psychiatry—A Systematic Review
Source: Front Psychiatry. 2020 Jan 16;10:963. doi: 10.3389/fpsyt.2019.00963 (PMC6976536; doi:10.3389/fpsyt.2019.00963)
Supplement: Supplementary file 1 [file DataSheet_1.docx]

Cochrane Library via Wiley January 11, 2018

Title: Pharmacological treatments in forensic psychiatry

| Search terms | | Items found |
| --- | --- | --- |
| Population: persons within forensic institutions/mentally ill criminals | | |
|  | [mh "forensic psychiatry"] or (([mh prisoners] or [mh crime] or [mh criminals] or [mh prisons]) **and** ([mh "mental disorders"] or [mh "schizophrenia spectrum and other psychotic disorders"] or [mh "child development disorders, pervasive"] or [mh "personality disorders"])) | 1162 |
|  | (offender* or criminal* or offending or offend* or forensic or incarcerate* or justice* or delinquent* or inmate* or correctional or prison* or (violent NEXT offence*) or reoffend* or (re NEXT offend*)):ti **and** (psychiatric* or mental* or "dual disorder" or "dual disorders" or psychotic* or psychosis or schizo* or autis* or asperger* or delusional or bipolar or "personality disorder" or "personality disorders" or borderline or antisocial or narcissistic* or "compulsive disorder" or "compulsive personality" or "dependent personality" or histrionic or paranoi* or negativistic or "behavior disorder" or "behavior disorders"):ti | 145 |
|  | ((forensic* NEAR/2 (psychiatr* or institution* or inpatient* or patient* or out-patient* or outpatient* or "clinical practice" or hospital* or treatment* or service* or ward* or mental* or facilit* or clinic* or neuropsych* or "personality disorder" or center* or unit*)) or ("forensic setting" or "forensic settings" or "forensic population" or "forensic populations") or ((hospital or hospitals) NEAR/3 ("maximum security" or "maximum securities" or "high-security" or "high securities" or medium or "low security" or "low securities" or "lower security" or "lower securities" or minimum*)) or ((maximum or high* or medium or low* or minimum or forensic*) NEXT secur*) or ((offender* or criminal* or offending or offend or forensic or incarcerate* or "juvenile justice system" or "juvenile justice systems" or delinquent* or inmate* or "correctional population" or "correctional populations" or "correctional setting" or "correctional settings" or "correctional facilit*" or "correctional institution*" or "correctional mental health" or prison* or "violent offence*" or reoffend* or "re offence" or "re offender") NEAR/4 ("mentally disorder" or "mentally disorders" or "mental disorder" or "mental disorders" or "mental disability" or "mental disabilities" or "mental health" or "mentally ill" or "mental illness" or psychotic* or psychosis or schizo* or autis* or asperger* or delusional or bipolar or "personality disorder" or "personality disorders" or borderline or antisocial or narcissistic* or "compulsive disorder" or "compulsive personality" or "dependent personality" or histrionic or paranoi*or negativistic or "behavior disorder" or "behavior disorders")) or ("secure psychiatric service" or "secure psychiatric services" or "secure psychiatric facility" or "secure psychiatric facilities" or "secure setting*" or "secure settings" or "secure hospital" or "secure psychiatric" or "secure environment" or "secure environments")):ti,ab,kw | 427 |
|  | 1 or 2 or 3 | 1482 |
| Intervention: antipsychotic agents | | |
|  | [mh "antipsychotic agents"] or [mh clozapine] or [mh risperidone] or [mh haloperidol] or  [mh chlorprothixene] or [mh chlorpromazine] or [mh aripiprazole] | 5813 |
|  | (antipsychotic* or clozapine or risperidone or haloperidol or levomepromazine or chlorpromazine or perphenazine or ziprasidone or flupentixol* or chlorprothixene or zuclopenthixol* or olanzapine* or zyprexa or quetiapine* or seroquel or aripiprazole or abilify or paliperidon* or asenapine* or lurasidone*):ti,ab,kw | 12920 |
| Intervention: mood stabilizers | | |
|  | [mh anticonvulsants] or [mh "valproic acid"] or [mh lithium] or [mh carbamazepine] | 3700 |
|  | ("mood stabilizer" or "mood stabilizers" or anticonvulsant* or anticonvulsive* or "neuroleptic drug" or "neuroleptic drugs" or "neuroleptic agents" or neuroleptics or lithium or "alproic acid" or depakine or valproate or carbamazepine or tegretol or lamotrigine):ti,ab,kw | 8982 |
| Intervention: benzodiazepines, zolpidem, zopiclone | | |
|  | [mh benzodiazepines] or [mh flunitrazepam] | 8004 |
|  | (benzodiazepine* or zolpidem or stilnoct or zopiclone or imovane or nitrazepam or flunitrazepam):ti,ab,kw | 6636 |
| Intervention: stimulants for ADHD | | |
|  | [mh methylphenidate] or [mh "atomoxetine hydrochloride"] or [mh "lisdexamfetamine dimesylate"] or [mh amphetamines] | 2532 |
|  | (methylphenidate or concerta or equasym or ritalin* or strattera or amphetamine* or metamina or dexamfetamine or attentin* or adderall) :ti,ab,kw | 3407 |
| Intervention: opiate substitution treatment/alcohol deterrents | | |
|  | [mh methadone] or [mh disulfiram] or [mh "alcohol deterrents"] or [mh naltrexone] or [mh "buprenorphine, naloxone drug combination"] or [mh buprenorphine] | 2693 |
|  | (methadon* or disulfiram or antabus or acamprosate or campral or alcohol deterrent or alcohol deterrents or naltrexon* or buprenorphine or lisdexamfetamine or attentin* or elvans*):ti,ab,kw | 5672 |
| Combined sets | | |
|  | 5 or 6 or 7 or 8 or 9 or 10 or 11 or 12 or 13 or 14 | 38318 |
|  | 4 and 15 | **176** CDSR/6  DARE/5  Central/  160  CRM/0  HTA/0  EED/5 |

The search result, usually found at the end of the documentation, forms the list of abstracts.

.ab. =Abstract

.ab,ti. = Abstract or title

.af.= All fields

Exp= Term from the Medline controlled vocabulary, including terms found below this term in the MeSH hierarchy

.sh.= Term from the Medline controlled vocabulary

.ti. = Title

/ = Term from the Medline controlled vocabulary, but does not include terms found below this term in the MeSH hierarchy

* = Focus (if found in front of a MeSH-term)

* or $= Truncation (if found at the end of a free text term)

.mp=text, heading word, subject area node, title

Embase via Elsevier January 11, 2018

Title: Pharmacological treatments in forensic psychiatry

| 1. Search terms | | Items found |
| --- | --- | --- |
| Population: persons within forensic institutions/mentally ill criminals | | |
|  | 'forensic psychiatry'/de | 12804 |
|  | ('offender'/exp or 'prison'/de or 'prisoner'/de or 'felony'/de or 'crime'/de) **and** ('mental patient'/de or 'mental disease'/de or 'psychosis'/exp or 'attention deficit disorder'/de or 'autism'/exp or 'personality disorder'/exp) | 9808 |
|  | ((forensic*) NEXT/2 (psychiatr* or institution* or inpatient* or patient* or out-patient* or outpatient* or "clinical practice" or hospital* or treatment* or service* or ward* or mental* or facilit* or clinic* or neuropsych* or "personality disorder" or center* or unit*)):ti,ab | 6785 |
|  | ("maximum security" or "high security" or "medium security" or "low security" or "minimum security" or "forensic security"):ti,ab | 1374 |
|  | ("secure psychiatric service*" or "secure psychiatric facilit*" or "secure setting*" or "secure hospital" or "secure psychiatric" or "secure environment*" or "forensic setting" or "forensic settings" or "forensic population" or "forensic populations"):ti,ab | 1514 |
|  | 1 or 2 or 3 or 4 or 5 | 25452 |
| Intervention: antipsychotic agents | | |
|  | 'neuroleptic agent'/exp or 'atypical antipsychotic agent'/exp | 259921 |
|  | (antipsychotic* or clozapine or risperidone or haloperidol or levomepromazine or chlorpromazine or perphenazine or ziprasidone or flupentixol* or chlorprothixene or zuclopenthixol* or olanzapine* or zyprexa or quetiapine* or seroquel or aripiprazole or abilify or paliperidon* or asenapine* or lurasidone*):ti,ab | 97865 |
| Intervention: mood stabilizers | | |
|  | 'anticonvulsive agent'/exp or 'lithium'/de | 408541 |
|  | ("mood stabilizer" or "mood stabilizers" or anticonvulsant* or anticonvulsive* or "neuroleptic drug" or "neuroleptic drugs" or "neuroleptic agents" or neuroleptics or lithium or "alproic acid" or depakine or valproate or carbamazepine or tegretol or lamotrigine):ti,ab | 114280 |
| Intervention: benzodiazepines, zolpidem, zopiclone | | |
|  | 'benzodiazepine derivative'/exp or 'zolpidem'/de or 'zopiclone'/de or 'nitrazepam'/de or 'flunitrazepam'/de | 204965 |
|  | (benzodiazepine* or zolpidem or stilnoct or zopiclone or imovane or nitrazepam or flunitrazepam):ti,ab | 47772 |
| Intervention: stimulants for ADHD | | |
|  | 'methylphenidate'/de or 'atomoxetine'/de or 'lisdexamfetamine'/de or 'amphetamine derivative'/exp | 103751 |
|  | (methylphenidate or concerta or equasym or ritalin* or strattera or amphetamine* or metamina or dexamfetamine or attentin* or adderall):ti,ab | 36315 |
| Intervention: opiate substitution treatment/alcohol deterrents | | |
|  | 'methadone'/de or 'disulfiram'/de or 'drugs used in the treatment of addiction'/exp | 122699 |
|  | (methadon* or disulfiram or antabus or acamprosate or campral or alcohol deterrent or alcohol deterrents or naltrexon* or buprenorphine or lisdexamfetamine or attentin* or elvans*):ti,ab | 15512 |
| Study type: randomized controlled trials, other trials and controlled studies | | |
|  | 'randomized controlled trial'/exp or 'controlled clinical trial'/exp or 'controlled study'/exp or 'major clinical study'/de or 'clinical article'/de or randomized:ti,ab or placebo:ti,ab or 'drug therapy':lnk or randomly:ti,ab or trial:ti,ab or groups:ti,ab | 12199890 |
| Limits | | |
|  | ([danish]/lim or [english]/lim or [norwegian]/lim or [swedish]/lim) |  |
| Combined sets | | |
|  | 7 or 8 or 9 or 10 or 11 or 12 or 13 or 14 or 15 or 16 | 854964 |
|  | 6 and 19 | 1256 |
|  | 6 and 19 and 17 and 18 | 843 |

The search result, usually found at the end of the documentation, forms the list of abstracts.

*OBS!
Sökdokumentationen räknas som arbetsmaterial och får inte spridas utanför projektgruppen innan rapporten publiceras. Om sökstrategin i sin helhet används i andra sammanhang (t.e.x vid publicerande av artikel) bör man hänvisa till den publicerade sökdokumentationen på* [*www.sbu.se*](http://www.sbu.se)

*OBS!
Sökdokumentationen räknas som arbetsmaterial och får inte spridas utanför projektgruppen innan rapporten publiceras. Om sökstrategin i sin helhet används i andra sammanhang (t.e.x vid publicerande av artikel) bör man hänvisa till den publicerade sökdokumentationen på* [*www.sbu.se*](http://www.sbu.se)

*OBS!*

*Sökdokumentationen räknas som arbetsmaterial och får inte spridas utanför projektgruppen innan rapporten publiceras. Om sökstrategin i sin helhet används i andra sammanhang (t.e.x vid publicerande av artikel) bör man hänvisa till den publicerade sökdokumentationen på* [*www.sbu.se*](http://www.sbu.se)

/de= Term from the EMTREE controlled vocabulary

/exp= Includes terms found below this term in the EMTREE hierarchy

/mj = Major Topic

:ab = Abstract

:au = Author

:ti = Article Title

:ti:ab = Title or abstract

* = Truncation

“ “ = Citation Marks; searches for an exact phrase

Medline via Ovid January 11, 2018

Title: Pharmacological treatments in forensic psychiatry

| 1. Search terms | | Items found |
| --- | --- | --- |
| Population: persons within forensic institutions/mentally ill criminals | | |
|  | exp "Forensic Psychiatry"/ or ((exp Prisoners/ or exp Crime/ or exp Criminals/ or "Prisons"/) **and** (Mental Disorders/ or exp "Schizophrenia Spectrum and Other Psychotic Disorders"/ or exp Child Development Disorders, Pervasive/ or exp Personality Disorders/)) | 53567 |
|  | (offender* or criminal* or offending or offend* or forensic or incarcerate* or justice* or delinquent* or inmate* or correctional or prison* or (violent adj1 offence*) OR reoffend* or re-offend*).ti **and** (psychiatric* or mental* or "dual disorder" or "dual disorders" or psychotic* or psychosis or schizo* or autis* or asperger* or delusional or bipolar or "personality disorder" or "personality disorders" or borderline or antisocial or narcissistic* or "compulsive disorder" or "compulsive personality" or "dependent personality" or histrionic or paranoi* or negativistic or "behavior disorder" or "behavior disorders").ti | 4533 |
|  | ((forensic* adj2 (psychiatr* or institution* or inpatient* or patient* or out-patient* or outpatient* or "clinical practice" or hospital* or treatment* or service* or ward* or mental* or facilit* or clinic* or neuropsych* or "personality disorder" or center* or unit*)) or ("forensic setting" or "forensic settings" or "forensic population" or "forensic populations") or ((hospital or hospitals) adj3 ("maximum secur*" or "high-secur*" or "high secur*" or medium or "low secur*" or "lower secur*" or minimum*)) or ((maximum or high* or medium or low* or minimum or forensic*) adj (secur*)) or (offender* or criminal* or offending or offend or forensic or incarcerate* or "juvenile justice system*" or delinquent* or inmate* or "correctional population*" or "correctional setting*" or "correctional facilit*" or "correctional institution*" or "correctional mental health" or prison* or "violent offence*" or reoffend* or re-offend*) adj4 ("mentally disorder*" or "mental disorder" or "mental disabilit*" or "mental health" or "mentally ill" or "mental illness" or psychotic* or psychosis or schizo* or autis* or asperger* or delusional or bipolar or "personality disorder" or "personality disorders" or borderline or antisocial or narcissistic* or "compulsive disorder" or "compulsive personality" or "dependent personality" or histrionic or paranoi* or negativistic or "behavior disorder" or "behavior disorders") or ("secure psychiatric service*" or "secure psychiatric facilit*" or "secure setting*" or "secure hospital" or "secure psychiatric" or "secure environment*")).ti,ab | 4065 |
|  | 1 OR 2 OR 3 | 56105 |
| Intervention: antipsychotic agents | | |
|  | exp Antipsychotic Agents/ or Clozapine/ or Risperidone/ or Haloperidol/ or  Chlorprothixene/ or exp Chlorpromazine/ or Aripiprazole/ | 124292 |
|  | (antipsychotic* or clozapine or risperidone or haloperidol or levomepromazine or chlorpromazine or perphenazine or ziprasidone or flupentixol* or chlorprothixene or zuclopenthixol* or olanzapine* or zyprexa or quetiapine* or seroquel or aripiprazole or abilify or paliperidon* or asenapine* or lurasidone*).ti,ab | 77017 |
| Intervention: mood stabilizers | | |
|  | exp Anticonvulsants/ or Valproic Acid/ or Lithium/ or Carbamazepine/ | 163041 |
|  | ("mood stabilizer" or "mood stabilizers" or anticonvulsant* or anticonvulsive* or "neuroleptic drug" or "neuroleptic drugs" or "neuroleptic agents" or neuroleptics or lithium or "alproic acid" or depakine or valproate or carbamazepine or tegretol or lamotrigine).ti,ab | 96964 |
| Intervention: benzodiazepines, zolpidem, zopiclone | | |
|  | exp Benzodiazepines/ or Flunitrazepam/ | 68106 |
|  | (benzodiazepine* or zolpidem or stilnoct or zopiclone or imovane or nitrazepam or flunitrazepam).ti,ab | 37987 |
| Intervention: stimulants for ADHD | | |
|  | exp Methylphenidate/ or exp Atomoxetine Hydrochloride/ or exp Lisdexamfetamine Dimesylate/ or exp Amphetamines/ | 45913 |
|  | (methylphenidate or concerta or equasym or ritalin* or strattera or amphetamine* or metamina or dexamfetamine or attentin* or adderall).ti,ab | 31881 |
| Intervention: opiate substitution treatment/alcohol deterrents | | |
|  | exp Methadone/ or exp Disulfiram/ or exp Alcohol Deterrents/ or exp Naltrexone/ or exp Buprenorphine, Naloxone Drug Combination/ or exp Buprenorphine/ | 27624 |
|  | (methadon* or disulfiram or antabus or acamprosate or campral or alcohol deterrent or alcohol deterrents or naltrexon* or buprenorphine or lisdexamfetamine or attentin* or elvans*).ti,ab | 27145 |
| Study type: randomized controlled trials and other trials (filter: Therapy -maximizes sensitivity)^[[1]](#footnote-1)^ and comparative study | | |
|  | (clinical trial.mp. or exp Clinical Trial/ or random:.mp. or tu.xs. or comparative study/) | 7041387 |
| Limits: | | |
|  | Limited to (danish or english or norwegian or swedish) |  |
| Combined sets | | |
|  | 5 or 6 or 7 or 8 or 9 or 10 or 11 or 12 or 13 or 14 | 462268 |
|  | 4 and 17 | 1080 |
|  | 4 and 15 and 16 and 17 | **811** |

The search result, usually found at the end of the documentation, forms the list of abstracts.

.ab. =Abstract

.ab,ti. = Abstract or title

.af.= All fields

Exp= Term from the Medline controlled vocabulary, including terms found below this term in the MeSH hierarchy

.sh.= Term from the Medline controlled vocabulary

.ti. = Title

/ = Term from the Medline controlled vocabulary, but does not include terms found below this term in the MeSH hierarchy

* = Focus (if found in front of a MeSH-term)

* or $= Truncation (if found at the end of a free text term)

.mp=text, heading word, subject area node, title

PsycInfo via EBSCO January 11, 2018

Title: Pharmacological treatments in forensic psychiatry

| 1. Search terms | | Items found |
| --- | --- | --- |
| Setting: forensic institution | | |
|  | DE "Forensic Psychiatry" or DE "Forensic Psychology" or DE "Mentally Ill Offenders" | 13474 |
|  | (DE "Crime" or DE "Criminal Behavior" or DE "Criminal Rehabilitation" or DE "Criminals" or DE "Juvenile Delinquency" or DE "Female Delinquency" or DE "Male Delinquency" or DE "Maximum Security Facilities" or DE "Correctional Institutions" or DE "Reformatories") **and** (DE "Mental Disorders" or DE "Schizophrenia" or DE "Acute Schizophrenia" or DE "Catatonic Schizophrenia" or DE "Childhood Schizophrenia" or DE "Paranoid Schizophrenia" or DE "Process Schizophrenia" or DE "Schizophrenia (Disorganized Type)" or DE "Schizophreniform Disorder" or DE "Undifferentiated Schizophrenia" or DE "Schizoaffective Disorder" or DE "Schizoid Personality Disorder" or DE "Schizotypal Personality Disorder" or DE "Schizotypy" or DE "Psychosis" or DE "Acute Psychosis" or DE "Affective Psychosis" or DE "Alcoholic Psychosis" or DE "Capgras Syndrome" or DE "Childhood Psychosis" or DE "Chronic Psychosis" or DE "Experimental Psychosis" or DE "Hallucinosis" or DE "Paranoia (Psychosis)" or DE "Postpartum Psychosis" or DE "Reactive Psychosis" or DE "Senile Psychosis" or DE "Toxic Psychoses" or DE "Autism Spectrum Disorders" or DE "Personality Disorders" or DE "Antisocial Personality Disorder" or DE "Avoidant Personality Disorder" or DE "Borderline Personality Disorder" or DE "Dark Triad" or DE "Dependent Personality Disorder" or DE "Histrionic Personality Disorder" or DE "Narcissistic Personality Disorder" or DE "Obsessive Compulsive Personality Disorder" or DE "Paranoid Personality Disorder" or DE "Passive Aggressive Personality Disorder" or DE "Sadomasochistic Personality" or DE "Schizoid Personality Disorder" or DE "Schizotypal Personality Disorder") | 6730 |
|  | TI(forensic*) or AB(forensic*) | 16058 |
|  | TX ((hospital) N3 (secur*)) | 1033 |
|  | TX ((maximum or high* or medium or low* or minimum or forensic*) W2 (secur*)) | 3382 |
|  | TX ((offender* or criminal* or offending or offend* or forensic or incarcerate* or justice* or delinquent* or inmate* or correctional or prison* or "violent offence*" or reoffend* or re-offend*) N2 ("mentally disorder*" or "mental disorder" or "mental disabilit*" or "mentally ill" or "mental illness" or psychotic* or psychosis or schizo* or autis* or asperger* or delusional or bipolar or "personality disorder" or "personality disorders" or borderline or antisocial or narcissistic* or "compulsive disorder" or "compulsive personality" or "dependent personality" or histrionic or paranoi* or negativistic or "behavior disorder" or "behavior disorders")) | 6959 |
|  | TX ("secure psychiatric service*" or "secure psychiatric facilit*" or "secure setting*" or "secure hospital" or "secure psychiatric" or "secure environment*") | 864 |
|  | 1 or 2 or 3 or 4 or 5 or 6 or 7 | 31506 |
| Intervention: antipsychotic agents | | |
|  | DE "Neuroleptic Drugs" or DE "Aripiprazole" or DE "Clozapine" or DE "Molindone" or DE "Nialamide" or DE "Olanzapine" or DE "Quetiapine" or DE "Reserpine" or DE "Risperidone" or DE "Spiroperidol" or DE "Sulpiride" or DE "Tetrabenazine" | 31137 |
|  | TX (antipsychotic* or clozapine or risperidone or haloperidol or levomepromazine or chlorpromazine or perphenazine or ziprasidone or flupentixol* or chlorprothixene or zuclopenthixol* or olanzapine* or zyprexa or quetiapine* or seroquel or aripiprazole or abilify or paliperidon* or asenapine* or lurasidone*) | 50055 |
| Intervention: mood stabilizers | | |
|  | DE "Mood Stabilizers" or DE "Carbamazepine" or DE "Lithium" or DE "Valproic Acid" | 11757 |
|  | TX ("mood stabilizer" or "mood stabilizers" or anticonvulsant* or anticonvulsive* or "neuroleptic drug" or "neuroleptic drugs" or "neuroleptic agents" or neuroleptics or lithium or "alproic acid" or depakine or valproate or carbamazepine or tegretol or lamotrigine) | 51718 |
| Intervention: benzodiazepines, zolpidem, zopiclone | | |
|  | DE "Benzodiazepines" or DE "Alprazolam" or DE "Chlordiazepoxide" or DE "Clonazepam" or DE "Diazepam" or DE "Flunitrazepam" or DE "Flurazepam" or DE "Lorazepam" or DE "Midazolam" or DE "Nitrazepam" or DE "Oxazepam" or DE "Hypnotic Drugs" | 17026 |
|  | (benzodiazepine* or zolpidem or stilnoct or zopiclone or imovane or nitrazepam or flunitrazepam) | 16040 |
| Intervention: stimulants for ADHD | | |
|  | DE "Methylphenidate" or DE "Amphetamine" or DE "Dextroamphetamine" or DE "Methamphetamine" | 17216 |
|  | TX (methylphenidate or concerta or equasym or ritalin* or strattera or amphetamine* or metamina or dexamfetamine or attentin* or adderall) | 19222 |
| Intervention: opiate substitution treatment/alcohol deterrents | | |
|  | DE "Methadone" or DE "Disulfiram" or DE "Naltrexone" or DE "Narcotic Antagonists" or DE "Buprenorphine" or DE "Acamprosate" | 11338 |
|  | TX (methadon* or disulfiram or antabus or acamprosate or campral or alcohol deterrent or alcohol deterrents or naltrexon* or buprenorphine or lisdexamfetamine or attentin* or elvans*) | 13202 |
| Limits | | |
|  | **Limiters** - Language: Danish, English, Norwegian, Swedish |  |
| Combined sets | | |
|  | *9 or 10 or 11 or 12 or 13 or 14 or 15 or 16 or 17 or 18* | *126422* |
|  | 8 and 22 and 21 | 532 |

The search result, usually found at the end of the documentation, forms the list of abstracts.

AB = Abstract

AU = Author

DE = Term from the thesaurus

MM = Major Concept

TI = Title

TX = All Text. Performs a keyword search of all the  database's searchable fields

ZC = Methodology Index

* = Truncation

“ “ = Citation Marks; searches for an exact phrase

1. http://hiru.mcmaster.ca/hiru/HIRU_Hedges_MEDLINE_Strategies.aspx [↑](#footnote-ref-1)
